# A Community-Based Participatory Action Research for Roma Health Justice in a Deprived District in Spain

**DOI:** 10.3390/ijerph16193722

**Published:** 2019-10-02

**Authors:** Daniela E. Miranda, Manuel Garcia-Ramirez, Fabricio E. Balcazar, Yolanda Suarez-Balcazar

**Affiliations:** 1 CESPYD, Coalition for the Study of Health, Power and Diversity, Department of Social Psychology, Universidad de Sevilla, Seville 41018, Spain; danimiranda@us.es; 2 Department of Disability and Human Development, University of Illinois at Chicago, Chicago, IL 60608, USA; fabricio@uic.edu; 3 Department of Occupational Therapy, University of Illinois at Chicago, Chicago, Il 60612, USA; ysuarez@uic.edu

**Keywords:** Roma, health inequities, antigypsyism, health justice, community-based participatory action research

## Abstract

Addressing health disparities and promoting health equity for Roma has been a challenge. The Roma are the largest disadvantaged ethnic minority population in Europe and have been the victims of deep social and economic injustices, institutional discrimination, and structural antigypsyism over many centuries. This has resulted in a much worse health status than their non-Roma counterparts. Current strategies based on ameliorative and top-down approaches to service delivery have resulted in paradoxical effects that solidify health disparities, since they do not effectively address the problems of vulnerable Roma groups. Following a health justice approach, we present a community-based participatory action research case study generated by a community and university partnership intended to address power imbalances and build collaboration among local stakeholders. This case study involved a group of health providers, Roma residents, researchers, Roma community organizations, and other stakeholders in the Poligono Sur, a neighborhood of Seville, Spain. The case study comprises four phases: (1) identifying Roma health assets, (2) empowering Roma community through sociopolitical awareness, (3) promoting alliances between Roma and community resources/institutions, and (4) building a common agenda for promoting Roma health justice. We highlighted best practices for developing processes to influence Roma health equity in local health policy agendas.

## 1. Introduction

Roma poor health outcomes are a manifestation of the prevalent marginalization and discrimination they continue to suffer [1]. It is estimated that between 12 and 15 million Roma currently live in Europe, making them the largest ethnic minority group on the continent as well as the poorest and the unhealthiest [2]. The World Health Organization (WHO) and the EU Fundamental Rights Agency (FRA) have denounced that 80% of European Roma have a life expectancy up to 15 years lower than the non-Roma population as a consequence of living in marginalized communities under the poverty threshold [3]. After 14 years of sustained intergovernmental political efforts aimed at overcoming these inequities, there is a general consensus that this scenario—far from improving—has worsened, and the inefficiency of initiatives is due in part to the underestimation of systemic “antigypsyism” embedded in European society [1]. Antigypsyism is a historically constructed, persistent complexity of customary racism against social groups identified under the stigma “gypsy”. “Gypsy” homogenizes these groups and incorporates social structures and violent practices focused on reproducing structural disadvantages [1,4,5,6,7]. Antigypsyism has forced Roma to endure on the margins of society while preventing them from accessing the fundamental opportunities and resources required to enjoy a healthy life.

The WHO urges for a health justice approach in order to create an ethic of public affairs where every individual—regardless of ethnic origin—has the resources to be as healthy as possible according to their personal values and traditions [8]. Therefore, initiatives are encouraged to focus on creating opportunities for Roma to engage in decision making regarding the allocation of resources [9]. We present a collaborative action-research process involving Roma residents, academic researchers, and health and social care providers aimed at developing health justice in Poligono Sur, a disenfranchised district in southern Spain with a high Roma population [10].

In this section, we provide a general overview of how antigypsyism jeopardizes Roma people’s health, the theoretical components for Roma health justice, and the methodological approach of the initiative. In the following section, we present a case study for Roma health justice in Poligono Sur, Seville (Spain). Finally, we present lessons learned and recommendations to incorporate this framework for future initiatives. For the purpose of this paper, Roma refers to people living in a specific neighborhood (i.e., Poligono Sur) who identify themselves as “Gitanos/as”. Roma is the term adopted by the Council of Europe to refer to ethnic groups such as “Roma, Sinti, Kale and related groups in Europe, including Travellers and the Eastern groups and covers the wide diversity of the groups concerned, including persons who identify themselves as Gyspies” [11], p. 4.

### 1.1. Antigypsyism and Roma Health Inequities in Spain

Antigypsyism (also Romaphobia) is “defined as a form of dehumanisation, because prejudice against the Roma clearly goes beyond racist stereotyping whereby the Roma are associated with negative traits and behaviour. Through dehumanisation, the Roma are viewed as less than human; and, being less than human, they are perceived as not morally entitled to human rights equal to those of the rest of the population” [11], p. 12.

Antigypsyism in Europe has been forged over centuries. Since their arrival in Europe, the history of Roma has been forged by persecution, systematically fleeing from slavery, widespread intolerance, expulsions, genocide, racist attacks, and punitive laws on their traditions and ways of life [12]. They have—and are—being constantly forced to move or to settle in segregated neighborhoods where they suffer from discrimination, poverty, and substandard living conditions [13,14]. In Spain, Roma are the largest ethnic minority—between 700,000 and 900,000—and more than 40% live in the southern Spanish region of Andalusia [15]. Spanish Roma have paradoxically oscillated between fascination and repudiation to the point that they were the most disliked group in Spain in comparison to other minority and impoverished groups (e.g., migrants, Muslims, homeless people) [16]. Modern day practices of expulsion continue to occur, such as patterns of gentrification that obligate their displacement, perpetuating spatial segregation, living in unsanitary settlements and insecure, and overcrowded living conditions [2,17,18].

Antigypsyism is also embedded within public service systems, including healthcare services. This is exemplified by accessibility barriers, negative attitudes of providers, exclusion from public health campaigns and programs, and absence of basic services in their neighborhoods [19]. National health surveys in 2014 reported worsening health conditions measured by indicators such as self-perceived health, chronic diseases, hearing and sight problems, dental cavities, obesity, and diabetes [20]. These surveys showed that an estimated 77% of Roma live in relative poverty, and their health status is significantly lower than that of low-income non-Roma. Roma health is also worse than non-Roma in all age segments [21]. The austerity measures implemented by the Spanish government from 2008 have exacerbated these health inequities while undermining measures taken to combat them [22].

Roma have developed a strong sense of agency in order to survive under these circumstances. This has been interpreted by non-Roma people as Roma’s inability to live “integrated” into mainstream society [23]. For example, given Roma’s values for extended family ties, it is typical for the extended family to be present and care for their sick loved ones when they are treated in healthcare services (e.g., primary health care centers, hospitals). Non-Roma Spaniards have a hard time accepting this cultural value. Dealing with a large number of extended family members makes communication between patient and providers challenging. This is commonly seen as unacceptable behavior by healthcare providers [24,25]. Daily interactions transform Roma protective factors into opportunities for discrimination in such a way that Roma are hostage to the antigypysist narratives, practices, and laws that are used against them [26]. Health care policies are habitually designed and implemented without Roma people’s input, in part due to the belief that they are incapable of achieving a prosperous life [6,8].

In 2011, the Spanish Ministry of Health adopted the European policy framework—known as National Roma Integration Strategies (NRIS)—to respond to these inequities [27]. This framework assumes that Roma health inequities are a “wicked challenge” due to their pervasive character, the lack of consensus about causes, difficulties developing collaboration and commitment among stakeholders, absence of an intersectoral approach, and the absence of Roma influence [19]. This framework underscores the need for Roma organizations to be involved in the design, the implementation, and the assessment of the initiatives. However, recent reports indicate that, while Roma organizations are under pressure to implement programs, their voices are not being heard; thus, they are unable to represent the voices of the most vulnerable Roma people, and therefore the programs being implemented are not recognized by Roma communities [28,29,30]. In the 2018 12th meeting of the European Platform for Roma Inclusion, it was concluded that specific programs implemented in partnership between healthcare institutions and Roma organizations aimed at reducing the high rate of obesity among Roma, improving sexual and reproductive health, or increasing campaigns to promote awareness for vaccination have not achieved—and in some cases have worsened—their desired outcomes [31]. To challenge antigypsyism structures and to address its consequences in Roma health, this paper proposes a health justice approach

### 1.2. Roma Health Justice

A Roma health justice (RHJ) approach would allow us to understand that (a) Roma health inequities are determined by the negative influence of antigypsyism over all social structures and (b) the lack of opportunities in sociopolitical participation is one of the most concerning consequences of pervasive antigypsyism [32]. Adopting a RHJ approach requires highlighting and mobilizing Roma capacities to lead in changes that follow the needs and the *interests* of the locality/neighborhood through: (a) strengthening Roma health assets; (b) developing capacity to influence among silenced Roma neighbors; (c) articulating Roma first-hand influential participation in community spaces; and (d) meaningful involvement of Roma in the reallocation of significant resources [8,33,34]. This approach requires specific community-based methodologies capable of building shared critical knowledge while inspiring sociopolitical change.

#### 1.2.1. Strengthening Roma Health Assets

An asset-based approach to health involves identifying strengths within the community and involving Roma in developing solutions to their health needs based on their own resources [35]. This approximation transcends traditional deficit-based approaches without the participation of Roma, thus developing systems in which Roma communities become dependent on professional and institutional resources [19]. Following community development literature, we define assets as individual, community, organizational, or institutional resources or characteristics that promote health and wellbeing for Roma [36]. Roma have shown resilience based on their traditions, family structures, sense of spirituality, solidarity, and commitment. These values have allowed them to remain cohesive in extremely adverse circumstances [37]. Over centuries of discrimination, Roma have maintained their collective mattering through insufflating their traditions, their collective values, and their artistic expressions [32]. This has contributed to an unbreakable capacity to build in-group identity and an extraordinary capacity of distinction from the out-group. In fact, the lack of a homeland has given them a strong ability to quickly transform the places where they arrive into their own; the lack of a state has led them to develop resilient standards for living together. The streets where they meet are important community spaces where they weave their social networks, and the households where they live become safe and empowering settings for children and elderly. For example, a recent study in Spain showed that the Evangelical church plays an important role for some Roma populations by providing a protective and supportive social environment [38]. These elements have a strong meaning for their living standards and—indeed—in the meaning of their health and in their culture of care [39]. The benefits of an asset-based approach highlights the capacity of Roma communities to guide change and reverse the misconception that they are helpless and dependent [35,40].

#### 1.2.2. Developing Capacity to Influence among Silenced Roma Neighbors

The RHJ approach strengthens an intersectional perspective capable of integrating the multiple axes of vulnerability that Roma suffer in impoverished neighborhoods [41]. Narrower approaches have not only led to the invisibility of the most disenfranchised groups but have even worsened their health outcomes. RHJ overcomes assuming that all Roma share the same experience and recognizes the complexity of discriminatory intersections and its effects by including the voices of those who suffer multiple sources of inequities [42]. In order to challenge dominant discourses, RHJ aims to strengthen broad and effective participation of usually silenced groups by deconstructing and reconstructing individual and group narratives [33,43,44,45,46,47]. Based on self-construction theory, this implies facilitating processes that empower Roma communities to redefine their individual and group experiences as a violation of their health rights and recognize their strengths to defend them [48]. RHJ develops leadership of silenced Roma ranks by promoting socio-political awareness of their health rights, identifying discriminatory experiences, becoming aware of their agency, and developing capacity to take action [45,49,50].

#### 1.2.3. Building Collaborative Capacity among Key Stakeholders

For Roma to influence change within their community, local key stakeholders who exert power over local resources and decision-making processes need to recognize them as legitimate stakeholders. Community organizing tactics that examine the power dynamics involved in contexts should be employed [34]. This consists of reexamining the role of mediating structures that maintain the status quo and institutions that comprise providers with negative attitudes towards Roma [51]. For example, at the organizational level, Roma community-based organizations (CBOs) are treated as mediating platforms between public institutions and silent Roma ranks. Roma CBOs assume responsibility of addressing Roma-needs but lack in dismantling dominant and antigypsyist narratives within prestigious organizational structures. The literature strongly proves that culturally sensitive providers can contribute to individual and community socio-political control to explicitly address discrimination [52,53]. Developing Roma sensitivity among non-Roma providers is required to deconstruct their unfair and oppressive narratives. This requires being aware that their performance could be biased by their social-cultural privileges while becoming necessary allies of initiatives led by Roma [54].

#### 1.2.4. Meaningful Involvement of Roma in the Reallocation of Significant Resources

An RHJ approach aims to develop alliances in order to promote collaboration between different sectors and encourage systemic agreements and strategies [44]. This builds a sustainable and shared decision-making platform of overarching health priorities at the local level that can quickly mobilize stakeholders and resources [55]. Following community development strategies, HJA creates opportunities for dialogue between multiple stakeholders, promotes the leadership of Roma neighbors, and develops social solidarity through shared critical knowledge [56]. Creating participatory structures gives opportunities to silenced groups to articulate their priorities and translate their local knowledge to influential Roma organizations who do have political opportunities during the policymaking process [57]. This involvement leads to safe and empowering community spaces to exert real influence on the initiatives and the policies that affect them [58].

### 1.3. Community-Based Participatory Action Reserach

An RHJ approach requires being implemented through community-based participatory action research (CbPAR). Understood as an instrument for social change in the struggle against oppression, we conceive CbPAR as “a process through which members of an oppressed group or community identify a problem, collect and analyse information, [identify allies and create alliances] and act upon the problem in order to find solutions and to promote social and political transformation” [59], p. 17.

The implementation of CbPAR processes requires the appropriate time, multiple strategies (e.g., participatory observation, testimonials, narratives, advocacy, art-based performances), multiple stakeholders, and the meaningful involvement of people with different capabilities in order to gain a deep understanding of geographic contexts, use of space, the role of community resources, etc. [60].

CbPAR has proven to be a suitable methodology in addressing health inequities of ethnic minorities. It has shown its capacity to identify and address problems related to human immunodeficiency virus (HIV) infections, nutrition, obesity and diabetes, gender violence, mental health conditions, drug abuse, cardiovascular diseases, families with children with disabilities, as well as health promotion among ethnic-based groups [58,61,62,63,64,65].

Therefore, CbPAR supports RHJ because it targets the promotion of the leadership of Roma guided by Roma needs and resources and driven by their strengths and cultural patterns, ultimately building among participants a sense of belonging, skill development, and meaningful engagement and leadership. This approach gives real power to Roma participants to instigate system changes while incorporating the best available scientific evidence [61]. Researchers act as critical friends, providing technical assistance, guidance, training, and partnership with communities as a way to develop local-based evidence that highlights valuable resources, identifies and prioritizes their community concerns, and advocates for them in local agendas.

## 2. Case Study

### 2.1. Background

#### 2.1.1. Community Context

There are an estimated 40,000 people living in the district of El Poligono Sur (PS), and approximately 20% identify themselves as Roma. Although there are no reliable data on the number of Roma living in the PS, as the census does not collect ethnic information, it is a well-known Roma enclave. In the 1960s, the Roma communities and families living in Triana, a historical and emblematic Roma neighborhood in Seville, were displaced to the PS as a result of gentrification and forced resettlements. PS is the poorest district in Spain, characterized by poor housing conditions, high unemployment rates, high rates of school absenteeism, and worse health status as compared to the rest of the city (Seville). Women have a life expectancy six years less than the rest of the population, and men have a life expectancy 10 years less [66]. These data are similar to those obtained by the Roma National Health Survey carried out in 2014 [67]. Today, the majority of Roma continue to live in the most precarious conditions and in the most excluded areas of the district.

PS has an extensive history of community-based activism that demanded from the City Council resources to improve the living conditions. As a result, in 2005, the City Council supported the development of a local plan [68]. This plan was supervised and implemented by a local public institution that promotes collaboration between organizations through intersectoral working groups that address health, education, housing, and employment. The local public institution has an important influence over local public services and community-based organizations, such as leading on coordination and collaboration. However, despite these efforts and the allocation of resources to PS, the Spanish National Institute of Statistics classifies PS as the most impoverished neighborhood in Spain [69].

A recent study evaluating the national Roma integration strategies health component in PS revealed that Roma populations are invisible in these local policies, plans, and participatory platforms [70]. For example, Escobar-Ballesta et al. (2018) [28] found that PS sexual and reproductive health programs focused on family planning have not been successful, in part because they ignore the Roma people’s voices and their value towards large families and moral constraints in sexual patterns. Local policies and programs are ignoring other at-risk contextual factors. These previous studies highlight that PS continues to implement strategies that are based on mechanisms of equality, thus excluding the most at-risk groups, such as Roma, living in the most vulnerable areas.

#### 2.1.2. The University-Community Partnership

This study was implemented by a university and community partnership, which followed the principles of mutual benefits and community engagement as articulated by Suarez-Balcazar, Harper, and Lewis (2005) [71]. University-community partnerships (henceforth partners) are considered reciprocal relationships in which university partners provide methodological tools for evaluation and identification of best practices as well as access to resources, while community partners provide in-depth knowledge of the community context, cultural sensitivity to decision-making processes, and access to hard-to-reach community members. The partnership of this study was composed by the Center of Community Action Research of the University of Seville (CESPYD, Coalition for the Study, Health, Power and Diversity; www.cespyd.org), which has experience developing and implementing action-research methods with communities. The community partner, FAKALI, (Federation of Roma Women’s Association), is a Spanish platform of Roma women (www.fakali.org) who have experience working directly with communities and advocating for Roma rights and political representation in the Roma State Council. In the case of Polígono Sur, FAKALI was participating in health working groups in order to ensure that Roma health was included in local agendas. CESPYD led a previous study that evaluated Roma health policies at the national level in which FAKALI participated. This past collaboration consolidated the relationship between CESPYD and FAKALI. Lessons learned from this experience highlighted the strengths of this type of partnership for future Roma health policy evaluations at the local level [72]. We refer to FAKALI as the community partner and CESPYD as the research partner in this paper.

### 2.2. Participants

The community partner’s initial contact with the local health policy coordinator, their participation in health working groups, and their experience with the primary healthcare center facilitated recruitment of health care providers. We invited providers from health working groups with semi-structured interviews and surveys (described below) to participate in the study. A total of eight sensitive providers from social services, primary healthcare centers, the public housing agency, and waste management services participated. These providers participated on the basis of their personal motivations and needs to improve their services through ensuring Roma-sensitive services. The providers’ roles were to participate in workshops that support Roma health justice, be a reference within their organizations for Roma health, recruit other providers to participate in the study, and nominate local Roma community members (henceforth “neighbors”).

Community partners and providers nominated Roma neighbors to participate in order to ensure a fair selection process. Research partners held face-to-face interviews with the nominees and invited neighbors to participate based on the following criteria: (a) identify themselves as Roma; (b) reside in the most impoverished areas of the district; (c) self-recognized as valuable members of Roma community; and (d) leadership capacity. A total of 20 neighbors were invited to create a parallel working group. A total of 15 neighbors participated in the study, 13 women and two men between the ages of 24–45. We intentionally chose neighbors from three of the most disenfranchised areas of the district in order to include multiple perspectives and reach the most silent groups. The Universidad de Seville and the Andalusian Health Council ethical board approved the implementation of this initiative, all participants signed informed content, and neighbors were given an economic compensation for their time in participating in this initiative.

### 2.3. Procedure

**Figure 1:**
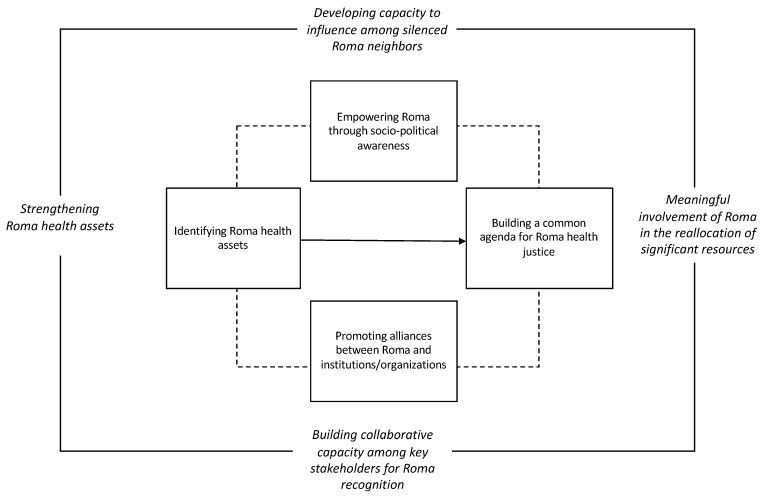
Community-based participatory action research process for Roma health justice at the local level.

As depicted in Figure 1, we designed a four stage community-based participatory action research process focused on: (a) identifying Roma health assets, (b) empowering Roma through sociopolitical awareness, (c) promoting alliances between Roma and institutions and organizations and, (d) building a common agenda for Roma health justice. This study took place from February 2016 to January 2017.

#### 2.3.1. Identifying Roma Health Assets

This was an iterative process targeted at identifying key stakeholders within the community. Key stakeholders included policymakers, representatives from public institutions, and Roma organizations. It was fundamental to identify Roma sensitive stakeholders in order to build a common language and purpose, to create spaces for constructive dialogue and reflection, and to develop systems of evaluation [73]. The objective of this stage was for partners and key stakeholders to build collective knowledge about the unfair Roma living conditions and the social determinants of Roma health inequities [65].

Initial semi-structured interviews with local providers were carried out in order to do a rapid community assessment of local Roma health and identify entry points into the community and potential providers to participate in the study workshops [74]. Interviews comprised the following questions: (1) “What experiences do you have working with the local Roma population?” (to better understand the background of each providers and their initial reactions towards Roma); (2) “Are there Roma-specific services?” (in order to identify existing resources and potential collaborators); and lastly, (3) “How would you describe the living conditions of local Roma population?” (to identify Roma and their relation to the most disenfranchised areas of PS). Two university partner researchers carried out eight interviews, which lasted 45–60 minutes and were recorded. Interviews were transcribed and triangulated with researcher’s field notes to consolidate information.

The university partner designed surveys to identify and understand the relationship of Roma neighbors to their local health care resources, to identify potential providers to participate in the study, and to assess overall Roma sensitivity in the community. During August and September of 2016, a total of 200 neighbor surveys were collected. See Appendix A for survey questions. Neighbors were asked in what area of the district they lived and to identify services frequented by type (educational, health, social services, etc.) and then to specifically evaluate the frequented services of their choice. Partners collaborated with neighbors to conduct surveys with other neighbors who identified themselves as Roma. Surveys were intentionally carried out in local meeting points with high Roma populations such as local street markets, catholic and evangelical churches, plazas, and bars [75].

Additionally, 40 provider surveys were collected with the objective to rapidly assess provider and organizations with Roma sensitivity. We used a set of indicators selected from the Community Cultural Competence instrument. This instrument was developed to assess the equity and the cultural competence and sensitivity of healthcare providers and organizations working in multi-ethnic communities [53]. At the organizational level, questions fall under the following dimensions: (a) values and goals of the organization (e.g., social justice is an important value in my organization), (b) accessibility of services (e.g., my organization actively identifies accessibility barriers specific to the Roma community), (c) sensitivity towards the Roma community (e.g., my organization evaluates Roma user satisfaction), and (d) participation (e.g., my organization includes participatory mechanisms for Roma). Personal level dimensions include: (a) personal value of work (e.g., my work is important to me), (b) personal level of adaptation (e.g., I can adapt my work for Roma needs), and (c) sensitivity towards Roma (e.g., I value and respect Roma and/or other groups). Appendix B shows both organizational and personal level questions answered on a 5-point Likert scale.

#### 2.3.2. Empowering Roma through Sociopolitical Awareness

This was a creative process that elicited higher-order thinking in which Roma discussed and reflected about power imbalances in their community lives, built critical knowledge around their health rights, and activated their involvement in addressing them [33,76]. To operationalize this process, Photovoice was facilitated by academic partners in order for Roma to identify concerns, share personal discriminatory experiences, and build self-determination to defend their health rights.

Photovoice workshops were designed to be spaces for reflection, dialogue, and gathering of local evidence. First, two 2 h workshops were implemented to discuss and share neighbors’ health concerns. Next, neighbors agreed on a health priority and took photographs to represent it. An innovative feature of our Photovoice method was converting everyday resources, such as the personal smartphone application *WhatsApp*, to be used as a tool for health promotion and community evaluation. Each neighbor chose one priority to photograph and sent relevant photographs through the *WhatsApp* group between sessions. Two 2 h sessions were designed to share their individual photographs with the larger group. We displayed photographs on the projector to instigate dialogue. In order to facilitate discussion and identify forms of discrimination that were affecting their daily lives, we utilized the Photovoice showed methodology of Wang and Burris (1997) [63,64,77]: (a) What do you see here?; (b) What is really happening here?; (c) How does this relate to our lives?; (d) Why does this concern, situation, strength exist?; (e) How can we become empowered through our new understanding; and (f) What can we do? Data analysis consisted of identifying themes with neighbors as proposed by Foster-Fishman, Law, Lichty, and Aoun (2010) [78]. This process included individual reflections to brainstorm local Roma health problems and group dialogue to analyze causes and effects in order to categorize photographs into overarching themes and to develop recommendations.

#### 2.3.3. Promoting Alliances between Roma and Community Resources/Institutions

We worked in parallel with health service providers from public institutions and community agencies (henceforth “providers”). The workshop objectives were to improve providers’ understanding of the Roma communities through Roma health awareness and discussion of local health challenges, to create a collaborative environment, and to collect and examine data [51].

Eight workshops were scheduled on providers’ availabilities and motivations to include Roma health as a priority within their work objectives. Based on problem-based learning methods, each session encouraged dialogue and reflection among participants and partners to explore Roma health inequities [79]. Partners prepared content to develop providers’ sensitivity, facilitated dialogue, and, at the end of each meeting, providers committed to a follow-up action. Research partners took field notes and developed workshop minutes to follow-up with providers.

#### 2.3.4. Building a Common Agenda for Roma Health Justice

This phase included community organizing and development strategies such as (a) a field visit of European health mediators for feedback; (b) planning a meeting between partners; (c) participating with providers and neighbors in order to consolidate their work towards Roma health justice; (d) sharing tools for community evaluation; and (e) negotiating problem definitions and potential solutions.

First, a field visit was organized in PS for health mediators from across Europe to share good practices and feedback and to develop recommendations to include Roma health justice in health mediation agendas. Next, a workshop was organized between partners, providers, neighbors, and other key stakeholders in order to share and negotiate priorities, resources, and actions. This new space for collaboration was a horizontal infrastructure that acted as an empowering community setting for negotiating local resources and increasing accountability among stakeholders [80]. Partners planned the workshop with the following content: (1) share evidence; (2) identify allies; (3) identify opportunities for Roma engagement; (4) plan for implementation and sustainability of actions. Research partners took field notes and developed workshop minutes to follow-up with providers.

### 2.4. Results

#### 2.4.1. Identifying Roma Health Assets

Interviews and surveys allowed us to understand PS context, identify Roma sensitive providers to participate in workshops, and raise awareness regarding significant Roma resources. Initial interviews from community partners’ existing contacts allowed for partners to understand providers’ concerns as professionals working in PS and illustrate the bigger picture of Roma living conditions from non-Roma providers’ perspectives. The respondents included: two social services coordinators, two center for addiction workers, two community-based organization coordinators, one rehabilitation center coordinator, and one local policy coordinator. As it is possible to see in Table 1, providers identified Roma as a significant part of PS but were implementing an equality model based on a provider–user relationship to neighbors, for example, health mediation programs by Roma organizations. Providers expressed how organizations were playing a role in health mediation but were not enough to access or understand the PS Roma community. Roma-specific actions were not included within their protocols despite being a majority of their users and the most challenging ones. Providers expressed their lack of knowledge in responding to Roma-specific needs and identified this gap in knowledge as a contributor/barrier to accessibility of services.

**Figure 2:**
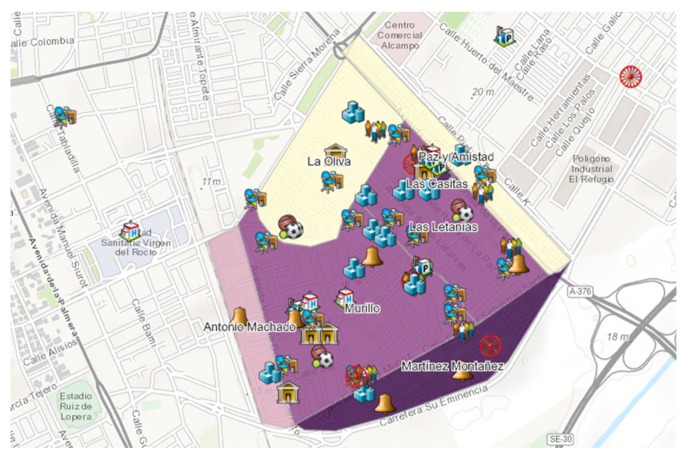
Screenshot of PS resources through a Roma sensitivity lens. This image shows density of Roma population (represented by darker shades) and identifies resources represented by type. For example, the bell icon represents faith-based organizations, blue centers represent educational services, and red circles represent Roma CBOs. This screenshot reveals that 80% of Roma live nearest two Evangelical churches and furthest away from formal health care services.

Figure 2 displays the density of Roma population represented by a color gradient separated by PS neighborhoods. The six neighborhoods were identified and defined in initial stakeholder interviews. The darker shades show a higher density of Roma population. Neighbor surveys revealed that 80% of Roma live in the worst areas of the district and confirmed that areas with high Roma population are hidden between large highways and a railroad track. Also, Figure 2 displays the services that were identified (represented by type of resources—healthcare services, faith-based organizations, Roma community-based organizations, etc.). Overall, areas with high Roma populations were lacking formal health services, and proximity to other community-based organizations (CBOs) were furthest from areas of high Roma density, except for a small Roma CBO and two evangelical churches.

Surveys revealed that public services such as the Housing Agency and the Employment Office scored lowest in both user satisfaction and organizational/provider Roma sensitivity. Informal services such as CBOs and faith-based organizations scored highest in user satisfaction and organizational/provider Roma sensitivity. Specifically, two Roma-led CBOs and one evangelical church located within the area with the highest Roma population scored highest. Roma-led CBOs provided activities for Roma youth in the area, mental health services such as family counseling and workshops to increase employability, and literacy workshops. Meanwhile, the Evangelical church was identified by more than half of the respondents and frequented by most more than twice a week. This specific church is led by Roma pastors and provides spiritual and emotional support. Educational settings that identified collaboration with Roma mediation programs and implemented programs adapted to Roma needs scored highest across both provider and neighbor surveys. Health mediation programs were implemented by larger Roma organizations that were not community-based. Surveys revealed that external places out of the Poligono Sur—such as parks and squares, bars, and shopping malls—were frequented and important in the community life of Roma neighbors. This wider use of services outside the neighborhood strongly suggests the relevance of open spaces in the Roma community life and an expression of the lack of these spaces and services in their surrounding spaces and neighborhoods.

In conclusion, provider interviews recognized that health mediation was useful in bringing Roma users to services; however, these respected health mediation programs in educational and healthcare settings were being implemented by Roma organizations without the collaboration of smaller Roma CBOs. Meanwhile, Roma-led faith-based organizations and CBOs assumed the role of providing resources that were recognized by the community as their own.

#### 2.4.2. Empowering Roma through Sociopolitical Awareness

**Figure 3:**
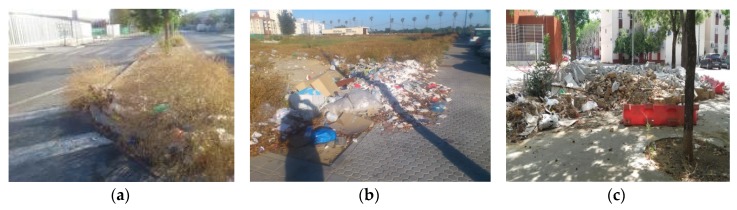
Unhealthy public spaces. (**a**) ¨There are around 40 or 50 homes and only one trash bin trash is left near the trees, the rats, mosquitos, insects, the situation is unbearable, the neighborhood is very dirty”. (**b**) “There used to be two football fields. People used to play football here. I would look out the window and watch. They were going to begin construction here and took away the field and now it has turned into this”. (**c**) “There are limited places for leisure. Perhaps three or four. They accumulate trash and things are left broken”.

**Figure 4:**
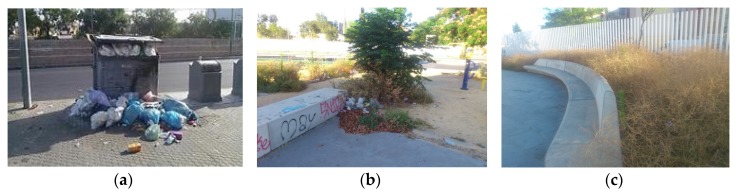
Neglect from public services. (**a**) “The local waste management office is only 20 meters away. They clean this area ever eight or nine days, the trash does not fit inside”. (**b**) “Here we can see the neglect of the city council in regards to parks and gardens. They never come to garden and trash just accumulates”. (**c**) “A big problem we have here in the Poligono Sur is the vegetation, here we do not have gardeners or anything with the consequences of fleas, rats, mosquitos, spiders, and it is incredible. You cannot see anything…you cannot see the plaza”.

**Figure 5:**
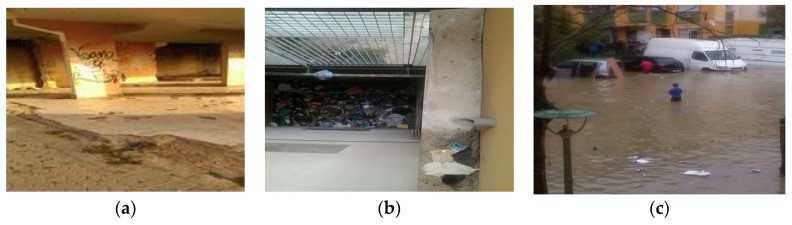
Unlivable housing conditions. (**a**) ”There was a rehabilitation plan, they took the people out for construction and it was left half done”. Despite local rehabilitation plans, “These buildings have been like this for ten years…homeless people and drug addicts occupy the building…when they are cold they build fireplaces and the building goes up in flames constantly”. (**b**) “There is a hole where a wash sink was that was torn out and now it is full of trash. Next door there are people who live with the trash and smell”. (**c**) This was a photograph a neighbor sent to the Whatsapp group after we finished the formal Photovoice sessions. After a day of rainfall in Sevilla, neighbors were unable to leave their homes because of the poor sewage system.

Photovoice workshops began with neighbors defining what health meant to them as a group. Neighbors then discussed and brainstormed a list of their community health concerns. The health concerns were then categorized in the following series of priorities: (a) plazas (open outdoor spaces), (b) lack of spaces and opportunities for leisure, (c) abandoned commercial spaces, (d) lack of trash bins near their homes, (e) absence of public services (e.g., public transportation), and (f) domestic risk factors. A total of 99 photographs were taken between May and June of 2016 that were representative of these environmental factors. Photographs were taken in different areas of PS near neighbors’ homes and surrounding communal spaces. Utilizing the photographs, partners facilitated discussion. Neighbors reflected on the discrimination and the abandonment they felt on a daily basis. Based on these causes, the photographs were categorized into three main overarching themes: (a) unhealthy public spaces, (b) neglect from public services, and (c) unlivable housing conditions. Figure 3, Figure 4 and Figure 5 are examples of each of these themes with photographs and neighbors’ narratives. For each theme, a series of recommendations/actions were proposed.

##### Unhealthy Public Spaces

The photographs presented under unhealthy public spaces showed the accumulation of trash everywhere, overgrown vegetation, construction left unfinished, sewage in spaces where children played, infestation of rats and insects near their homes, and commercial spaces that have been left in poor conditions. Figure 3 below represents photographs and narratives of unhealthy public spaces. Neighbors then developed the following recommendations/actions: (a) increase the number of waste bins, (b) increase frequency of waste collection, (c) raise awareness among neighbors to maintain clean spaces, and (d) improve the installations of commercial spaces.

##### Neglect from Public Services

Neighbors expressed an overall sense of abandonment from public services such as the waste management, parks and gardening, mail, public transport, and the local policy committee responsible for coordinating actions in the district. The conversation oscillated between blaming other neighbors for the poor conditions of common spaces to the complete neglect from those responsible of providing services in the neighborhood. It was highlighted that, in parts of the neighborhood where non-Roma lived, the living conditions were drastically different. Figure 4 below represents photographs taken in different public spaces in PS and narratives of unhealthy public spaces. Neighbors then developed the following recommendations/actions: (a) petition for an increase in the frequency of trash pick-up, public transport, and gardening, (b) request a local post office, and (c) implement city bike stations.

##### Unlivable Housing Conditions

The housing conditions of the areas where participating neighbors and most other Roma neighbors lived were the worst areas of the district. Government funded housing was abandoned, and projects had been left halfway done. Infrastructure (e.g., elevators, mailboxes, sewage systems, burnt homes) was left broken. Figure 5 below represents photographs and narratives of unhealthy public spaces. Neighbors then developed the following recommendations/actions: (a) restore housing conditions and (b) assess and fix the sewage system.

These results and recommendations were to be shared to the group of providers in a joint workshop described in Section 2.4.4.

#### 2.4.3. Promoting Alliances between Roma and Community Resources/Institutions

Based on needs and gaps detected in the survey (see 2.4.1.), partners were responsible for improving providers’ understanding of the Roma communities through raising awareness regarding antigypsyism, discussion of local health challenges, creating a collaborative environment, and collecting and examining data [54]. Topics discussed followed recommendations from the Spanish National Health Mediation network [81]. Each workshop ended with commitments from providers such as attending the next meeting, identifying two influential Roma neighbors, identifying other health providers as allies, and organizing a meeting to be held at the end of the study with Roma neighbors. Workshops were not recorded due to the sensitive issues being addressed. University partners took field notes and consolidated main ideas prior to developing minutes (logistics, content, and commitments) for each workshop and were then transferred back to providers.

As shown in Table 2 below, initial provider reactions were blaming Roma culture for their poor health outcomes. As the workshops progressed and new information was provided, providers’ narratives transformed into the identification of contextual factors (education, housing, employment) and the healthcare system exclusion of Roma as factors influencing Roma poor health outcomes. Lastly, providers committed to collaborating with Roma neighbors in a joint workshop in order to design actions forward.

#### 2.4.4. Building a Common Agenda for Roma Health Justice

A meeting was organized in PS between mediators from across Europe (Bulgaria, Romania, Macedonia, Spain), partners, neighbors, providers, and Roma CBOs. This consisted of sharing good practices between Roma health mediators from different European countries (Belgium, Bulgaria, Frane, Moldova, Romania, Slovaki, Spain, and Ukraine). The meeting was an opportunity for participating local providers and neighbors to share and disseminate the process and the work they had been doing. A general consensus of the meeting was that, in order to move forward in improving Roma health, it was necessary to transform the provider–user model that mediation programs apply towards processes that empower Roma populations living in marginalized communities. This was an important contribution for the wider-European agenda for Roma health [82]. (For more information: https://eea.iom.int/sites/default/files/publication/document/Equi-Health-Final-report.pdf).

Finally, a meeting was held at the local primary healthcare center. A total of 21 participants attended the meeting, comprising five neighbors, eight providers, and eight members of the university-community partnership. All providers that Roma had previously identified as relevant and important for them, representatives of social services, primary healthcare centers, the public housing agency, waste management services, and the chair of the Local Public Authority of Poligono Sur were invited. Partners facilitated discussion and ensured a climate for neighbors as protagonists of the meeting. For example, one neighbor reflected that the services that were available were not recognized by the neighbors and shared Photovoice results with providers in order to develop a common, shared understanding of the health concerns that were important to them. Providers who had been working in the neighborhood had not attributed health outcomes to the environmental issues that neighbors shared. Existing services were not recognized or utilized by neighbors: “There are a lot of services in PS but the people do not know them or do not know how to use them”. In response, providers recognized that local plans did not reflect the complexity of the context: “It [local policy plan] is a small, reduced vision of the dynamics in the PS.” This has caused the development of well-intentioned initiatives to not be sustainable over time. Neighbors expressed their sense of burnout: “We can all agree, that all of the people are tired of unfulfilled promises that begin and never finish.” Overall providers felt a need to work with neighbors to transform the living conditions and improve their own services: “Without the PS people, we will not change anything, it is necessary to create strategies with neighbors.”

Table 3 depicts the contents of the meeting, the collaborative set of proposals, and the outcomes of meeting negotiations. For example, during the identification of potential allies to address health concerns, neighbors and providers committed to identifying other neighbors and providers in working towards a collective goal, and this led to exchanging contact information between neighbors and providers.

We present results by short-term outcomes and actions directed towards structural changes. Short-term refers to tangible actions that were seen on the ground, while structural changes refer to actions incorporated at the institutional and organizational level.

A short-term outcome following this meeting was that partners continued funding by the Open Society Foundations *Road4Health* (2016–2019) initiative in which interested participants—health providers and neighbors—maintain collaboration and are consolidating these initial spaces for formal local coalitions in PS and other neighborhoods with high Roma populations. Participating neighbors have continued working in this direction by involving other neighbors, developing other strategies to collect evidence (i.e., surveys and interviews), disseminating results within their neighborhood, and gaining support from organizations and institutions. Local community-based organization *Estudio 41013* collaborated with the research team as a community expert and incorporated a health justice approach into their initiatives. *Estudio 41013* organized meetings with pastors from faith-based organizations and representatives from small Roma community-based organizations in order to begin collaborating in future initiatives.

In regards to structural changes, the primary healthcare center director supported partners in meeting with the Sevilla healthcare district directors to support institutionalizing workshops within the healthcare districts continuing education programs in order to raise awareness and identify strategies to support Roma neighbors in defending their health rights [54]. With the support of the community partner and the healthcare district, research partners formalized a continuing education program in the university for the following year. This continuing education program’s overall objective was to develop advocacy capacity among a group of Roma neighbors to incorporate a health justice approach in their respective communities. Health care providers have been supportive throughout the follow-up discussions and have maintained a collaborative relationship with both university and community partners. Following this study, partners formalized their relationship by signing a collaboration agreement with the University of Seville, and this included Roma health in academic agendas.

## 3. Discussion

The overall purpose of this paper was to implement a Roma health justice approach for transformative change in a deprived neighborhood with a high Roma population. We presented a case study of a community-based participatory action research process (CbPAR) that intended to increase sociopolitical control in order to deconstruct structural antigypsyism in local health care settings. We can work towards restoring Roma rights by developing individual and collective Roma sociopolitical awareness about discriminatory experiences. Through the Photovoice methodology, neighbors were given the opportunity to articulate their concerns individually, engage in dialogue around their rights as citizens to enjoy healthier living conditions, and to then advocate together for their rights in front of a group of providers. Our study provides lessons learned for future CbPAR to advance towards healthcare systems that recognize the rights of the diversity within our society.

Our findings confirm that current ameliorative interventions that focus on providing services or assistance may paradoxically contribute to reinforcing negative attitudes towards the Roma and attributing problems to a homogenized group instead of recognizing the multiple forms of discrimination that affect them [32]. For example, providers’ discourse was contrasted by their commitment when neighbors shared Photovoice results regarding unhealthy public spaces coinciding with health provider’s concerns. Photovoice results showed that neighbors were being ignored by public services and therefore challenged providers’ existing perceptions of Roma as a helpless community. This act of recognition or making Roma voices heard—both individually and collectively—shifted the traditional user–provider relationship to that of Roma as an equal collaborator that is capable of defining policy and interventions that directly affect community needs.

During the identification of resources, we generated evidence that distinguished between resources with institutional prestige and resources that engaged Roma communities while exposing institutions and organizations perpetuating Roma health inequities. For example, providers had difficulty accessing different layers of the Polígono Sur Roma community, yet smaller community-based organizations were able to have active participation and high satisfaction from them. Faith-based organizations, for example, the Evangelical church, provide services that are defined and sustained by the community themselves. These types of services are employing community-based approaches that include neighbors’ voices in designing resources, ensuring collective ownership and creating a network built on trust. Larger organizations and institutions should engage the Roma community from the beginning in order to gain a new sense of trust and make services their own. Future work should include collaboration on the ground with those resources recognized by Roma communities and develop a relationship based on mutual benefits and trust. Including Roma communities only as users of services perpetuates mistrust, lack of engagement, and discriminatory attitudes that maintain health inequities [39]. Gaining a real understanding of the Roma community context can stimulate change among providers’ attitudes and practices towards Roma and organizational equity [53]

Our initiative also highlights that Roma organizations have to ensure the voices of at-risk Roma without replacing them to advocate for health rights and to ensure that diverse and real needs are met. This challenge is complicated by organizations’ reliance/dependence on funding by governments whose plans tend to maintain the status quo. However, by engaging at-risk Roma, we can challenge these mechanisms as a form monitoring and accountability [83]. Including health justice principles in interventions that recognize Roma community knowledge enables us to dismantle asymmetrical power relations and deconstruct essentializing Roma health inequities [61]. Roma health justice requires evolving from efforts that focus on purely remedial solutions towards transformative policy changes that bring about systemic change and the balancing of power dynamics. Our CbPAR experience instigated Roma community members’ engagement in prioritizing local agendas and negotiating local resources. In this vein, we must build the capacity of Roma organizations to include Roma health justice approaches in their current interventions. Community-based organizations should develop policy-influencing skills and identify allies that can translate their collective knowledge to the policymaking arena in order to ensure access and quality of health services [65,84]. This would create less distance between influential spaces and hard-to-reach Roma communities.

Finally, our case study presents a set of limitations and challenges in regards to CbPAR. First, we specifically worked with Roma Kalo. We suggest that upcoming studies diversify and explore with other ethnic groups since CbPAR processes require sensitivity to complex contexts in which new community settings are formed [85,86]. As external experts, we acted as catalysts that instigated the process of shaping new relationships and redefining old relationships by organizing workshops between multiple stakeholders. Intersubjective dialogue and bidirectional learning processes between researchers, providers, neighbors, and other stakeholders can improve problem framing for influential policy changes [52]. However, this requires research and community partners to build relationships with different stakeholders of the community, which entails a large investment in time. Challenges regarding time can influence momentum and expectations and contribute to an overall sense of burnout among participants. Working with public services means that we depend on outside influences such as changes in funding, institutional protocols, and governments, which can then mark the time and the sustainability of these efforts. Another challenge is the history of collaboration between community stakeholders, which includes competition for similar resources. This can create fragmentation between resources and duplicity of services, which in turns makes the entry point into communities segmented and favor coverage for one part of the community. We suggest reinforcing alliances with university researchers who are not competing for the same resources. University-community relationships are optimal as they can reinforce each other to obtain both private and common goals for real social changes to social justice. University access to resources and evidence-based knowledge complement organizations’ experiential knowledge. In turn, this can ensure transparency and evaluation by documenting outcomes and revisiting health justice values.

## 4. Conclusions

A health justice approach requires us to respect and highlight these community characteristics, utilizing community codes as sources of collaboration and not as barriers that create weak alliances and lack of trust. Lessons learned from this experience can be transferred to other contexts with communities with other Roma ethnic communities. Despite Polígono Sur being a neighborhood with a long history of activism and the subject of university research of Roma organizations and community-based organizations, our initiative created new and empowering community settings that promoted Roma leadership [80]. Initiatives as the one described in this paper require the development of trusting relationships, the inclusion of all local stakeholders as experts, and participatory analyses that revisit policy commitments. Locally-based coalitions, action groups, and tasks should be recognized as legitimate actors.

These mixed methods allowed us to access legitimized Roma community leaders and learn to respect community entry points, time-frames, and processes in order to build trust within the community to build effective alliances and a true sense of collaboration. This was reflected in the commitment to continue working collaboratively between neighbors, university partners, and other community stakeholders. By instigating new alliances and collaboration between multiple community stakeholders in spaces for equal collaboration, it was possible to negotiate priorities and resources to be shared towards a collective goal. Our findings suggest that a community-based participatory action research model follows a Roma health justice approach by organizing new local settings that give a voice back to neighbors to readjust community agendas and resources based on real priorities with the support of a group of sensitive providers. In this direction, we believe that future initiatives are obligated to incorporate processes that engage Roma communities in leading meaningful efforts that influence equitable policy changes.

## Figures and Tables

**Table 1:** Sample quotes from stakeholder interviews for Poligono Sur (PS) rapid assessment.

Question	Quotes
What experiences do you have working with the local Roma population?	“We try to work with the Roma community because it is a priority in the Poligono Sur, and we understand that they have particular needs […] we are conscious that we lack mediation to better understand them, it is challenging.” “The ‘I am a social victim’ is a persistent problem of the population here. ‘By being a gypsy I have a right to everything’, and this is not the reality. Because there are non-Roma people who have much greater needs and we have to be provider in a wide spectrum. We are familiar with the Roma handicaps and obviously need to help […]”
Are their Roma-specific services?	“There are many local Roma non-governmental organizations however they are exclusive to only part of the Roma community so their services are not accessible to everyone who could be taking advantage of them” “[…] coordination is good with all community-based organizations, but many Roma organizations do mediation. When there is a real problem [with Roma] there is a lot of personal involvement then there can be no impartiality”
How would you describe the living conditions of local Roma population?	“There are many houses that are overcrowded, meaning that 3 or 4 generations are living together. They have done extensions to the original house […] they are looking for, as it is logical and normal, a way to survive.” “And here that happens very often […] many children with substance abuse problems use the pension [of elderly family member] for consumption.” “Because although these are apartment building, we consider them as vertical settlements […]”

**Table 2:** Content of provider workshops with excerpts from research partner’s field notes.

Modules	Topics	Field Notes of Provider Discussion
History and culture of Roma population	Roma history Cultural identity Social demographics	Providers attribute Roma health problems to culture and health practices, homogenizing group needs.
Roma health	Health diagnostics Health inequities	Providers begin to discuss marginalization as the main problem, questioning initial attribution to culture. Neglect of public spaces as a general concern for health issues.
Roma health and Spanish healthcare system	Cultural differences between Roma and healthcare system Structure and coverage of the healthcare system National Roma integration strategies health component	Blaming individualized habits of Roma. Structural issues are identified (e.g., lack of provider continuity in healthcare center) and no available resources to improve services for Roma. Providers did not have knowledge about Roma health policies.
Strategies to facilitate intervention	Peer mentoring Mediation Cultural competences*	Providers identified their own good practices when responding to local Roma needs. Identified frustrations due to lack of organizational support and the need to adapt their practices to local needs.
Health promotion with Roma community	Health education for Roma community Health accompaniment Intersectoral work Community networking Roma participation	Agreed that collaboration with Roma organizations and Roma neighbors was an action that could help improve local health outcomes.

**Table 3:** A workshop was held between neighbors and health providers. During this workshop, partners organized content and participants developed a series of proposals that were negotiated into the agreements/recommendations presented below.

Content	Neighbor Proposal	Agreement/Recommendations
(1) Share evidence (resource map and Photovoice)	Increase number of trash bins Raise awareness among others regarding clean spaces Improve frequency and quality of public service	Develop plan to meet with waste management representatives Providers offering navigational support of different institutions and resources within the community
(2) Identify allies	Commit to working with health providers and other Roma	Exchange of contact information between Roma and providers
(3) Identify opportunities for Roma engagement	Open channels for collaboration with local institutions	Invitation to participate in local health working groups by local policymaker
(4) Plan for implementation and sustainability actions	Offer support as Roma advocates within institutions and organizations	Follow-up meeting Continued funding from the Open Society Foundations Roma Initiatives Office

## Appendix A

**Table A1:** Roma neighbor survey for resource identification and evaluation.

Dimension	Questions
Use of community resources	How often do you attend these services (list of formal and informal services provided)
Level of satisfaction with services	I feel respected
	My opinions are taken into consideration
	I have the opportunity to participate in activities
	Activities with Roma are organized
	I feel understood
	I have problems receiving services
	I am satisfied with the service I receive
	Professionals are ready to work with Roma community
	It is easy to commute to from Poligono Sur

## Appendix B

**Table A2:** Organizational and health care provider survey for local Roma equity.

Level	Dimension	Questions
Organizational	Values and goals of the organization	Social justice is an important value in my organization My organization provides opportunities for Roma well-being
	Accessibility of services	My organization actively identifies accessibility barriers specific to Roma community My organization provides mechanisms to overcome these barriers
	Sensitivity towards Roma community	My organization evaluates if its practices are Roma-sensitive My organization registers Roma-specific information My organization provides Roma-specific training My organization adapts its practices to Roma needs My organization evaluates Roma-user satisfaction My organization provides its providers with Roma-specific resources
	Participation	My organization includes participatory mechanism for Roma Providers can influence within the organization My organization responds to Roma complaints and/or proposals My organization participates in Roma community activities My organization provides health mediation/mediation services My organization has formal relations with other organizations (if so, which ones)
Personal	Personal value of work	My work is important to me My role is important for organizational functioning
	Personal level of adaptation	I can adapt my work for Roma needs My organization provides me with information specific for adapting work with Roma My organization provides me with Roma-specific training I am competent when providing services to Roma I have support of other community resources I show appreciation towards Roma when providing services
	Sensitivity towards Roma	I value and respect Roma and/or other groups I effectively communicate with Roma users
